# A common optical approach to thickness optimization in polymer and perovskite solar cells

**DOI:** 10.1038/s41598-021-84452-x

**Published:** 2021-03-02

**Authors:** Olga D. Iakobson, Oxana L. Gribkova, Alexey R. Tameev, Jean-Michel Nunzi

**Affiliations:** 1 Frumkin Institute of Physical Chemistry and Electrochemistry of the Russian Academy of Sciences, 31 bld.4 Leninsky Prosp., Moscow, Russian Federation 119071; 2 National Research University Higher School of Economics, 20 Myasnitskaya Str., Moscow, Russian Federation 101000; 3 Department of Chemistry, Department of Physics, Engineering Physics and Astronomy, Queen’s University, Kingston, ON K7L-3N6 Canada

**Keywords:** Materials science, Materials for energy and catalysis, Solar cells, Optics and photonics, Applied optics, Solar energy and photovoltaic technology

## Abstract

The structure of experimentally designed solar cells was optimized in terms of the photoactive layer thickness for both organic bulk heterojunction and hybrid perovskite solar cells. The photoactive layer thickness had a totally different behavior on the performance of the organic and hybrid solar cells. Analysis of the optical parameters using transfer matrix modeling within the Maxwell–Garnett effective refractive index model shows that light absorbance and exciton generation rate in the photoactive layer can be used to optimize the thickness range of the photoactive layer. Complete agreement between experimental and simulated data for solar cells with photoactive materials that have very different natures proves the validity of the proposed modeling method. The proposed simple method which is not time-consuming to implement permits to obtain a preliminary assessment of the reasonable range of layer thickness that will be needed for designing experimental samples.

## Introduction

Solar cells (SCs) attract researchers attention as promising renewable energy sources. Two prospective types of so-called third generation SCs are currently under intensive investigation. First one is based on organic photoactive materials, which advantages include mechanical flexibility, light weight, and low fabrication cost into large-area devices^1–3^. The largest reported power conversion efficiency in organic solar cells is obtained using the bulk-heterojunction (BHJ) concept^1^. Bulk heterojunctions provide not only high surface contacts for charge separation, but also an interpenetrating network for efficient charge transport^1^. Second type of SCs that recently succesfully entered into the field of photovoltaics is based on metal–organic compounds with the perovskite structure. Perovskite materials are characterized by large absorption coefficients, relatively high carrier mobilities, long carrier lifetime, as well as simple fabrication process, which result in the development of highly efficient perovskite solar cells^4^.

One challenge in modern solar energy is the development of cost-effective devices with facile device structure optimization. Optimization requires proper understanding of the physical processes that underlie device performance^5^. There has been several efforts to understand the device operation mechanism of organic SCs. The effect of variables such as morphology, temperature, light intensity, and optical absorption on the device performance, and primarily on the short circuit current characteristics, has been studied extensively^1,6^.

In addition to experimental approaches in which optimization sometimes proceeds through trial and fail, attempts to analize device performances through simulation models has shown some success^1–3,7^. There are two general approaches in SC simulation that take into account optical (incoupling of the light to the multilayer stack)^1–3,7^ and electrical (extraction of charges)^2,3^ parameters of the device materials, and sometimes combinations of both^6,8^. However, electrical models can be quite combersome computationally^2^.

Influence of the optical effects on the performance of SCs is usually derived from the device design features since the thickness of thin films in SCs is generally smaller than the wavelength of the incident light^6^. The objective of optical modelling and simulation is to calculate the light absorption and exciton creation based on the materials properties and structure of the solar cells^6^. As shown by optical modelling^9^, the thickness optimization of each functional layer in SCs (photoactive active layer, electron transport layer and hole transport layer) would facilitate the performance improvement. Several models were implemented to analyze light absorbtion. The transfer matrix model has revealed fruitful in the calculation of optical interference, absorption, transmission and reflection in thin film devices, although only for those constructed from homogeneous and isotropic materials with flat interfaces^9^. Most polymeric layers without texturing meet these conditions^6^. For instance, Pettersson et al.^10^ used the transfer matrix formalism to calculate the absorbed optical energy in an organic multilayer structure, with results that agreed with the experimental data. However, the transfer matrix modeling may fail in predicting the parameters of perovskite solar cells owing to their microcrystalline structure and large index of refraction. Optical modeling was also recently applied to the optimization of the photoactive layer thickness in hybrid CH_3_NH_3_GeI_3_ perovskite SCs^4^.

In the present work, we successfully applied known optical simulation methods to analyze the performance of solar cells built from completely different materials. A newly engineered hole transport material superior than commonly used PEDOT-PSS: a polyaniline complexed with a polyacid, was employed in this study. As a limited number of works was devoted to a direct comparison of experimental and modelling results, the question whether it is possible to extend a given model to other materials is still open. Moreover, there were no comparative study investigating SCs based on alternative photoactive materials with hole transport material other than the commonly used PEDOT-PSS from both experimental and theoretical point of views. Full aggrement between simulated and experimental results allows us to conclude that the simple model can be used as a general predictive tool to optimize devices constructed from different materials. This simple approach offers the opportunity to rapidly test and validate novel device concepts. It avoids the lengthy fabrication of large sets of prototypes which is usually required for an experimental optimization^3^.

## Experimental part

Solar cells (SC) designed here are based either on an organic or an hybrid photoactive layer (PAL), with the structure: ITO (indium tin oxide) on glass (anode)/ hole transporting layer (HTL)/ photoactive layer (PAL)/ electron transporting layer (ETL) / Al (cathode).

ITO (100 nm) covered glass with 15 Ω/square sheet resistance (Kintec) served as anode. The glass support is thick enough to be insensitive to interference effects^9^. A drop-casted film of polyaniline complex with poly(2-acrylamido-2-methyl-1-propanesulfonic acid) (PANI-PAMPSA) obtained as described earlier^11,12^ was used as HTL. The comprehensive characterization of this material was previously published^11,12^.

For the organic PAL, a chlorobenzene mixture of regioregular P3HT polymer (4002-EE; Rieke Metals) and fullerene derivative PC_71_BM electron-acceptor (SES Research) (1:0.8 wt.) was used. Details of the PAL preparation can be found in^12^. The organic PAL thickness was varied by tuning the speed and time of centrifugation (1500 vs 900 & 2000 rpm and 60 vs 60 & 45 s). The LiF electron transporting layer (0.9 nm) and Al cathode (80 nm) were deposited on the PAL by thermal evaporation under ~ 10^–6^ mbar vacuum in an Auto500 Edwards Evaporator^13^.

The hybrid perovskite SC was based on a methylammonium lead iodide (MAPbI_3_) PAL. A N, N-dimethylformamide mixture of methylammonium iodide (Dyesol) and lead iodide (AlfaAesar) at different concentrations was deposited on the HTL by centrifugation (5000 rpm, 30 s). The MAPbI_3_ perovskite was formed by the method described earlier^14^. A Fullerene C_60_ electron transporting layer (40 nm), a 2,9-dimethyl-4,7-diphenyl-1,10-phenanthroline hole blocking layer (7 nm) and Al cathode (80 nm) were deposited on the PAL by thermal evaporation under ~ 10^–6^ mbar vacuum in an Auto500 Edwards Evaporator.

Thickness of all layers was determined using a KLA-Tencor D-100 Profiler. Photovoltaic performances were studied inside a glovebox using a Keithley 2400 SMU under the AM1.5 illumination of an Oriel solar simulator (Xe lamp 150 W, Newport Corp.).

Light propagation through the organic and hybrid SCs was studied by modeling the photocurrent action spectra using one-dimensional 2 × 2 optical transfer matrix method^10^. The method consists in representing the SC as a set of flat layers. Each one is characterized by its thickness and complex refractive index $\tilde{n} = \eta + i\kappa$, within the Maxwell–Garnett model^1^.

The following parameters were assessed from modeling: light absorbance in the PAL, the Shockley–Queisser limit at the real SC efficiency and the exciton generation rate in the PAL were assessed from modeling. Light absorption in the PAL was determined by:

1 $$ \mathop \smallint \limits_{{\lambda_{down} }}^{{\lambda_{up} }} A\left( \lambda \right) \cdot S_{\lambda } \cdot d\lambda $$

where ${\uplambda }$ is the wavelength, A(${\uplambda }$) is absorption coefficient, ${\text{S}}\left( {\uplambda } \right)$ is radiation power density normalized under AM1.5G condition with $\int^{\infty}_{0} S\left( \lambda \right)d\lambda = 1$. Shockley–Queisser limit at the real SC efficiency was determined following^15^:

2 $$ 10^{ - 9} \cdot \mathop \smallint \limits_{{\lambda_{down} }}^{{\lambda_{up} }} \frac{A\left( \lambda \right)}{{h \cdot c}} \cdot \lambda \cdot E_{g} \cdot S_{\lambda } \cdot d\lambda $$

where $E_{g}$ is the band gap, $h$ the Planck’s constant, $c$ the speed of light in vacuum. Exciton generation rate in the PAL was determined following^16^:

3 $$ G = \mathop \smallint \limits_{0}^{d} G\left( z \right)dz = \mathop \smallint \limits_{0}^{d} (\mathop \smallint \limits_{0}^{\infty } S\left( \lambda \right)\gamma \left( \lambda \right)\frac{\lambda }{hc}Q_{\lambda } \left( z \right)d\lambda )dz $$

where $d$ is the PAL thickness, $G\left( z \right)$ the exciton rate at normal incidence at coordinate z, $\gamma \left( \lambda \right)$ is photon to exciton conversion efficiency and $Q_{\lambda } \left( z \right)$ is the average energy flow dissipation.

Complex refractive indices were determined by analysis of the spectroscopic ellipsometry data obtained either experimentally using a UVISEL2 elipsometer for ITO glass, PANI-PAMPSA and MAPbI_3_ or from the literature for the BHJ PAL^17^, C_60_^18^, and Al^19^. Measured optical parameters provide a more accurate optical simulation^1^. Simulation was performed in the 360 nm to 800 nm spectral range.

## Results and discussion

Light harvesting as well as the density of generated excitons in the PAL vary along the layer thickness, which affects the short-circuit current (*J*_*sc*_) and in consequence the power conversion efficiency (PCE). Optimization of the functional layer thickness is therefore one of the routes to increase the PCE^3,4,7,20^. However, values of the optimal thickness will be specific to each particular system within a selected SC structure, and of the materials used for the PAL. However, provided that the processes undelying device physics are the same for different PALs, it is expected to find similar explanations to the observed dependences. Among the abundance of scattered exprimental data, no general model taking into account the physical parameters of the layers forming the SCs was proposed. Such model would make it possible to predict the dependence of the device efficiency on the layer thickness and thus to establish the optimal thickness range. We have studied the influence of PAL thickness on the power conversion efficiency (PCE) with two entitely different photoactive materials. One of them is the widely studied P3HT-PC_71_BM polymer composition which forms a bulk heterojunction (BHJ) in the PAL. The other one is the intensively studied methylammonium lead iodide (MAPbI_3_) perovskite which is an organic–inorganic (hybrid) microcrystalline material.

Influence of the P3HT-PC_71_BM and of other BHJ PAL thickness on the PCE of devices with a conventional PEDOT-PSS HTL was experimentally studied elsewhere^1,2,21–27^. The widespread use of PEDOT-PSS can be explained by its low-temperature solution processability, sufficiently high hole mobility from the conjugated structure, mechanical flexibility, and the ability to form thin films with improved film forming compared to small-molecule hole transport materials^28^. However, PEDOT-PSS easily aggregates forming damageable defects in the film morphology^29^. Moreover, such polymeric materials are relatively expensive and can increase the overall manufacturing cost of SCs^28^. In the search for hole transport materials made from low-cost conjugated polymers to achieve economical and efficient SCs, researchers turned to polyaniline, which is characterized by a low-cost monomer as well as an easy synthesis, reasonably high conductivity, high transmittance, and environmental stability. However, the use of conventional PANI results in a device with poor photovoltaic parameters^28^. We recently developed a polyaniline complex with a polyacid (PANI-PAMPSA), which is obtained as an aqueous dispersion via oxidative chemical polymerization of aniline in the presence of commercial PAMPSA^11^, with no further complicated purification process. Similar to PEDOT-PSS, PANI-PAMPSA is a solution-processable polymeric material which thin layer possesses mechanical flexibility and high transmittance in the visible range for solar applications. Moreover, when PANI-PAMPSA is used as an HTL, it carries some advantages over PEDOT-PSS, like the absence of an heterophase formation in dispersion during storage and the hygroscopicity of the layer, a reasonable electrical conductivity^30,31^, and stability of the electrical and optical properties for at least 2 years^32^. Moreover, organic SCs with the same structure bearing an HTL made of either PANI-PAMPSA or PEDOT-PSS showed similar performances^33^.

Table 1 shows the dependence of the device parameters, *J*sc and PCE, on the P3HT-PC_71_BM thickness for SC with a PANI-PAMPSA HTL. The thinnest P3HT-PC_71_BM layer is limited to 35 nm owing to the necessity of obtaining a continuous uniform thin film. The thicker the PAL, the larger *Jsc* and *PCE*. PCE increases more than twice within the studied range (Table 1). Further increase of the PAL thickness leads to a dramatic increase of the layer roughness. Moreover, while increasing the layer thickness would result in enhanced light harvesting, it also restricts charge extraction to the electrodes owing to the limited diffusion length of charge carriers which induces recombination losses^2,23,24,34^. Therefore, with PAL thicknesses larger than 100 nm, most generated charge carriers recombine before reaching the electrode^2^, which is the limit of validity of the proposed optical simulation.

**Table 1:** Dependence of the short-circuit current density (*Jsc*) and power conversion efficiency (*PCE*) on the P3HT-PC_71_BM thickness.

P3HT-PC_71_BM thickness, nm	*Jsc* (mA/cm^2^)	PCE (%)
35	4.07	1.3
55	5.85	1.9
95	8.82	3.0

Thickness of the perovskite absorber is also an important parameter contributing to optimize solar cell performances^4^. A completely different picture was found for MAPbI_3_ based SCs (Table 2) with the PANI-PAMPSA complex also used as an HTL. There is not a clear dependence of the average performance parameters on the PAL thickness for the MAPbI_3_ devices (Table 2). The PCE ranges between 7 and 9% with about 8% variation. Variation of the perovskite layer thickness in the studied 350–500 nm range does not significantly affect the PCE. It is worth noting that the PCE in the studied devices corresponds to the reported analogues in the literature^28,29^.

**Table 2:** Dependence of the short-circuit current density (*Jsc*) and power conversion efficiency (*PCE*) on the MAPbI_3_ thickness.

MAPbI_3_ thickness (nm)	*Jsc* (mA/cm^2^)	PCE (%)
350	15.00	7.6
370	17.08	8.2
390	14.62	8.0
420	15.30	9.0
450	14.04	7.1
470	15.96	8.4
490	17.62	8.3

The transfer matrix method was applied to identify the physical origin of the observed dependences^2,7,23,25,26,35^, based on the optical energy absorbed in the multilayer structures^10^. The method assumes that the materials in the component layers are nonmagnetic and isotropic^10^. Despite widespread use of optical transfer matrix methods for multilayer optical structures. the question about the possibility to extend a given model to simulations of SCs with microcrystalline PAL materials remained open. Moreover, the comparative study of the thickness dependence of P3HT-PC_71_BM and MAPbI_3_ based SCs with a PANI-PAMPA complex based HTL is presented for the first time.

The following processes generally take place during sunlight-to-energy conversion process in SC: (a) light absorption and excitons generation, (b) exciton diffusion to the interface and exitons dissociation at the interface, (d) free charge carriers transport to the electrodes^7^. Therefore, among the parameters that can be extracted from the calculation (see, for example, ref.^9^), the following parameters are usually enought for the assessment of SC performance: light absorbance in the PAL, the Shockley–Queisser limit at the real SC efficiency and the exciton generation rate in the PAL.

**Figure 1:**
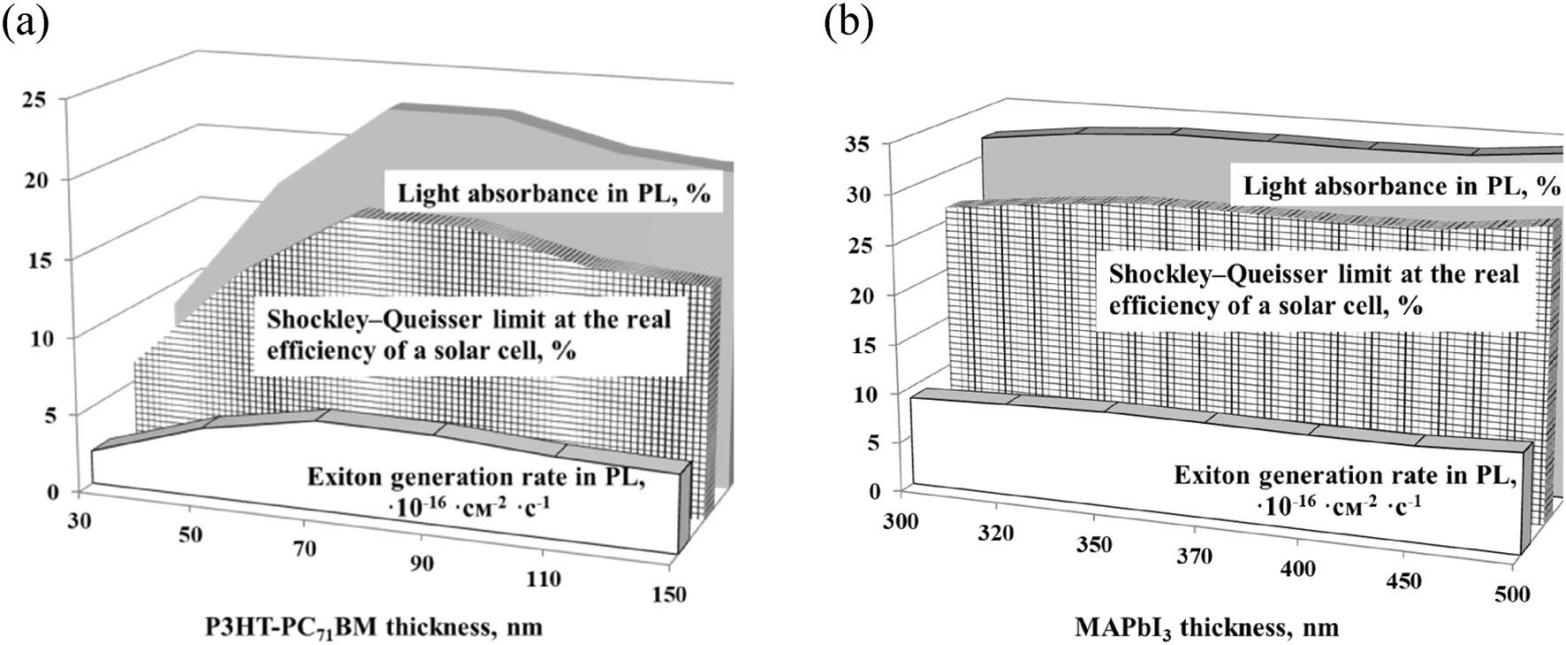
Calculated light absorbance in PAL, Shockley–Queisser limit at the real SC efficiency and exciton generation rate in the PAL for varied P3HT-PC_71_BM (**a**) and MAPbI_3_ (**b**) thickness.

Figure 1 shows the calculated optical parameters as a function of the PAL thickness for P3HT-PC_71_BM (a) and MAPbI_3_ (b). As seen in Fig. 1, the optical parameters demonstrate similar dependences within one type of PAL used. For the P3HT-PC_71_BM based PAL, the sharp increase of the calculated optical parameters with the increase of the PAL thickness till ~ 80 nm is followed by their slight decrease to a plateau level. Hence, the maximum PCE of the experimentally designed SC is likely to be explained by the combination of the highest rate of exciton generation and the maximum light absorption in the PAL at 90–100 nm thickness.

Oppositely, changes in the light absorbance, Shockley–Queisser limit at the real SC efficiency and exciton generation rate in the MAPbI_3_ based PAL with the increase of the PAL thickness are negligible. Therefore, the experimentally observed weak dependence of the PCE on the thickness of the perovskite PAL in the 350 to 500 nm range is explained by minor changes in both the light absorbance and the exciton generation rate.

The results obtained through modelling of the SC optical parameters agree with the obtained experimental data for both types of PAL. Therefore, we could demonstrate that the same model concepts are applicable to assess the range of optimal parameters of SC functional layers with different types of PAL. Obviously, the range of accessible layer thicknesses is governed by the thin film formation method. However, within the accessed experimental range, modelling with account of the functional layers’ optical parameters proves very efficient for specifying the range of thicknesses that ensure the maximum device efficiency.

## Conclusion

The matrix transfer method was used to explain theoretically the experimental effect of the thickness on the optical parameters of the layers and on the performance of SCs. We showed that the approach can be applied equally to the analysis of amorphous donor–acceptor systems and of microcrystalline structures. All of these were built with a water-dispersible polyaniline-polyacid complex as the hole transporting layer.

Although there has not been any pevious comprehensive study on the nature of the optical effects that are general enough to model SCs with completely different photoactive materials, our results show that optical modeling based on the transfer matrix method is applicable to solve the case. It provides an efficient method to validate proof-of-concepts regardless of the nature of the photoactive layers. We suggest applying the simulation method as a general predictive tool for the rapid testing and validation of novel devices constructed from different materials.
